# Non-High-Density Lipoprotein Cholesterol on the Risks of Stroke: A Result from the Kailuan Study

**DOI:** 10.1371/journal.pone.0074634

**Published:** 2013-09-10

**Authors:** Jianwei Wu, Shengyun Chen, Yong Zhou, Chunxue Wang, Anxin Wang, Qian Zhang, Xiang Gao, Haitao Hu, Shouling Wu, Xingquan Zhao

**Affiliations:** 1 Department of Neurology, Beijing TianTan Hospital, Capital Medical University, Beijing, China; 2 Department of Neural Stem Cell Transplantation, The General Hospital of Chinese People's Armed Police Forces, Beijing, China; 3 Channing Laboratory, Department of Medicine, Brigham and Women’s Hospital and Harvard Medical School, Boston, Massachusetts, United States of America; 4 Department of Nutrition, Harvard University School of Public Health, Boston, Massachusetts, United States of America; 5 Department of Medicine, Perelman School of Medicine, University of Pennsylvania, Philadelphia, Pennsylvania, United States of America; 6 Department of Cardiology, Kailuan Hospital, Hebei United University, Tangshan, China; University of Oxford, United Kingdom

## Abstract

**Aims:**

To prospectively explore the association between non-high-density lipoprotein cholesterol (non-HDLC) and the risks of stroke and its subtypes.

**Methods:**

A total of 95,916 participants (18-98 years old; 76,354 men and 19,562 women) from a Chinese urban community who were free of myocardial infarction and stroke at baseline time point (2006-2007) were eligible and enrolled in the study. The serum non-HDLC levels of participants were determined by subtracting the high-density lipoprotein cholesterol (HDLC) from total serum cholesterol. The primary outcome was the first occurrence of stroke, which was diagnosed according to the World Health Organization criteria and classified into three subtypes: ischemic stroke, intracerebral hemorrhage, or subarachnoid hemorrhage. The Cox proportional hazards models were used to estimate risk of stroke and its subtypes.

**Results:**

During the four-year follow-up, we identified 1614 stroke events (1,156 ischemic, 416 intracerebral hemorrhagic and 42 subarachnoid hemorrhagic). Statistical analyses showed that hazard ratios (HR) (95% Confidence Interval: CI) of serum Non-HDLC level for total and subtypes of stroke were: 1.08 (1.03-1.12) (total), 1.10 (1.05-1.16) (ischemic), 1.03 (0.96-1.10) (intracerebral hemorrhage) and 0.83 (0.66-1.05) (subarachnoid hemorrhage). HR for non-HDLC refers to the increase per each 20 mg/dl. For total and ischemic stroke, the risks were significantly higher in the fourth and fifth quintiles of non-HDLC concentrations compared to the first quintile after adjusting the confounding factors (total stroke: 4^th^ quintile HR=1.33 (1.12-1.59); 5^th^ quintile HR = 1.36 (1.15-1.62); ischemic stroke: 4^th^ quintile HR =1.34 (1.09-1.66); 5^th^ quintile HR = 1.53 (1.24-1.88)).

**Conclusions:**

Our data suggest that serum non-HDLC level is an independent risk factor for total and ischemic stroke, and that higher serum non-HDLC concentrations are associated with increased risks for total stroke and ischemic stroke, but not for intracerebral and subarachnoid hemorrhage.

## Introduction

Stroke is a major healthcare problem and a serious economic burden to society. In China, stroke is the leading cause of death and long-term disability. Currently, more than 7,000,000 individuals suffer from stroke, and there are 2,000,000 individuals with newly diagnosed stroke each year [1,2].

Atherosclerosis and lipoprotein disorders have been indicated as critical pathological changes during the development of stroke [3]. Non-high-density lipoprotein cholesterol (non-HDLC) is composed of several atherogenic lipoproteins, including very-low-density lipoprotein (VLDL), low-density lipoprotein (LDL), intermediate-density lipoprotein (IDL), and lipoprotein (a). The US National Cholesterol Education Program Adult Treatment Panel guideline III (NCEP-ATP III) has set goals for non-HDLC after the achievement of LDL cholesterol (LDLC) goals in patients with elevated triglycerides [3]. Recent studies have demonstrated that the predictive value of non-HDLC in coronary heart disease (CHD) incidence is similar to or better than LDLC [4–7]. With respect to stroke, studies have been conducted to investigate the relationship between non-HDLC and risk of stroke. Studies reported that elevated serum TC or LDLC concentrations were associated with increased risk of ischemic stroke but reduced risk for intracerebral hemorrhage (ICH) stroke [8]. In contrast, in the other two Japanese cohort studies no association between non-HDLC and stroke was found [9,10]. A cohort study on Chinese population recently found that the level of non-HDLC was a predictive factor for ischemic stroke [11]. In summary, previous reports regarding the relationship between non-HDLC and the risk of stroke remain highly controversial. Here we reported a large prospective cohort study with Chinese population to test if serum non-HDLC is associated with risks of different subtypes of stroke.

## Materials and Methods

### Study design and population

**Figure 1:**
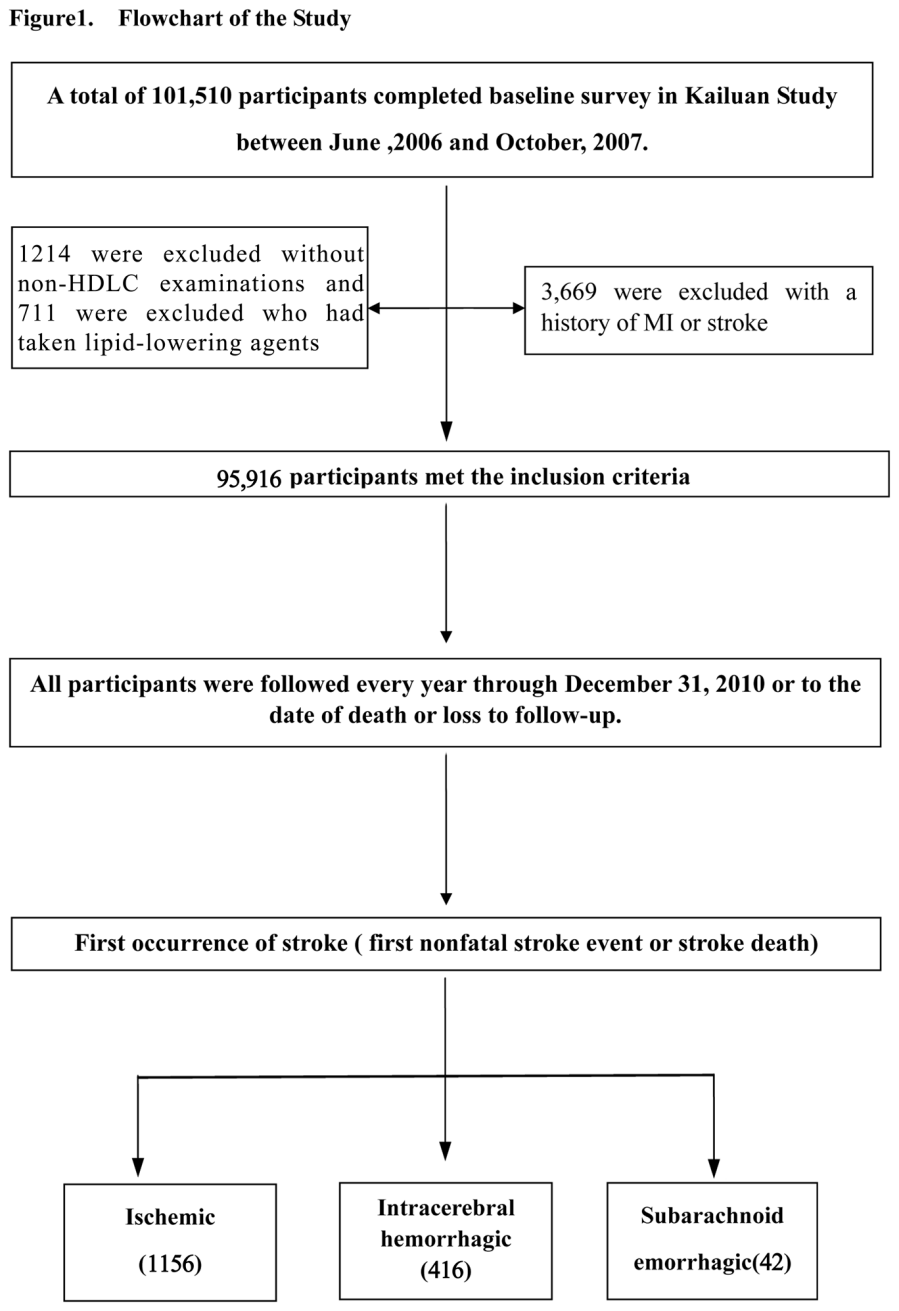
Flowchart of the study.

The Kailuan study was a prospective cohort based in Kailuan community in Tangshan city, which is a large and littoral modern city located in the southeast of Beijing. The detail information about Kailuan Study has been described previously [12–14]. From June 2006 to October 2007, all the 155,418 residents (including the retired) in the community were invited to participate. The inclusion criteria were: (1) aged 18 years or older; and (2) provided a written informed consent. In total, 101,510 (65.31%) participants (81,110 men and 20,400 women, aged 18-98 years old) were recruited into the Kailuan study. The participants enrolled in our study were mainly the mining employees of the Kailuan group with a significantly higher male to female ratio (4:1) than the general Chinese population. All the participants received questionnaire assessments, clinical and laboratory examinations, which were conducted by medical professionals in the 11 designated hospitals in Kailuan community. In this study, the following participants were excluded: 3,669 participants who had history of myocardial infarction (MI) and/or stroke at baseline, 1,214 participants who did not receive non-HDLC measurement (or whose serum non-HDLC level was not measured), and 711 participants who had taken lipid-lowering agents. Eventually, a total of 76,354 men and 19,562 women were included (Figure 1) in the analyses. The study was performed according to the guidelines of Helsinki Declaration and was approved by the Ethics Committees of Kailuan General Hospital, and Beijing Tiantan Hospital.

### Measurement of Non-HDLC level at baseline

Blood samples were collected from an antecubital vein of participants in the next morning after overnight fast (>8 hours) and then transferred into vacuum tubes containing EDTA (Ethylene Diamine Tetraacetic Acid). All the blood samples were processed and analyzed using an auto-analyzer (Hitachi 747; Hitachi, Tokyo, Japan) at the central laboratory of the Kailuan General Hospital.

Level of total cholesterol (TC) in serum was measured using endpoint test method. HDLC and LDLC were measured with direct test method. Triglyceride (TG) was measured by GPO method (inter-assay coefficient of variation < 10%; Mind Bioengineering Co. Ltd, Shanghai, China). Non-HDLC level was determined by subtracting serum HDLC from serum TC.

### Assessment of potential covariates at baseline

Information on demographic variables (e.g., age, sex, and education) was collected via questionnaires. The education level was categorized as “college or higher”, “middle school”, or “illiteracy or primary school”.

Information on smoking and physical activity was also collected via questionnaires. Weight and height were measured during the interview and body mass index (BMI) was calculated as weight (kg)/height (m)^2^. BMI was classified as ideal if it was <25 kg/m^2^. Systolic Blood pressure (SBP) and Diastolic Blood pressure (DBP) were measured twice in the seated position using a mercury sphygmomanometer. The average of two readings was used in the analyses.

### Assessment of incident stroke

The participants were followed up by face-to-face interviews at every two-year routine medical examination until December 31, 2010 or to the event of interest or death. The follow-ups were performed by hospital physicians, research physicians, and research nurses who were blinded to the baseline data. The outcome information for the participants without face-to-face follow-up was obtained by referring to death certificates from provincial vital statistics offices, discharge summaries from the 11 hospitals, and medical records from medical insurance.

The primary outcome was the first occurrence of stroke; either the first nonfatal stroke event or a stroke death. A nonfatal stroke was defined as a focal neurological deficit of sudden onset and vascular mechanism that lasted >24 hours. Stroke was diagnosed according to the World Health Organization (WHO) criteria [15] combined with brain computed tomography (CT) or magnetic resonance (MR) confirmation, and classified into three main types: ischemic stroke, intracerebral hemorrhage, or subarachnoid hemorrhage. The criteria were consistent across all participating hospitals. All stroke records were reviewed by two independent stroke specialists. If instances of disagreement in a single case, the final evaluation was made by the event adjudication committee. All the stroke outcomes were validated by the Data Safety Monitoring Board and Arbitration Committee for Clinical Outcomes.

### Statistical analysis

The participants were classified into 5 groups according to quintiles of serum non-HDLC level. In addition, medians and proportions of potential risk factors for stroke among these 5 groups were calculated. Continuous variables between groups were compared by Variance analysis, while categorical variables were analyzed by Chi-Square test. The raw and adjusted hazard ratios (HRs) with 95% confidence intervals (CIs) were calculated from the Cox proportional hazards model. There were 11 hospitals participating in the current study. Thus, the cox proportional hazard model with a sandwich covariance matrix as a random effect was used to account for the potential effects due to different treatment centers. Other potential confounders included age, gender, BMI, hypertension, diabetes, smoking and drinking status (‘never’, ‘former’, ‘occasional’, or ‘daily’), physical activity status (‘very active’, ‘moderately active’, or ‘inactive’), HDLC, and triglyceride. A trend test was used to examine the relationship between different quartile levels of serum non-HDLC and the risk of stroke. Outcome assessors and statisticians were independent and were blinded to the participants’ baseline information. All statistical analyses were performed with the SAS software, version 9.2 (SAS Institute, Cary, NC, USA). All CIs were estimated at the 95% level and signiﬁcance was set at a P value of <0.05 (2-sided).

## Results

### Baseline non-HDLC levels and characteristics of other variables for study participants

In our study, all participants were followed up to the event of interest or death through multiple ways. For participants who took the every two-year routine medical examination, they were followed up by face-to-face interviews by hospital physicians and nurses. For those without face-to-face interviews, their follow up information was obtained by checking death certificates from provincial vital statistics offices, discharge summaries from the 11 designated hospitals, or medical records from medical insurance companies. However, we were did not collect the information in this study regarding the proportion of the cohort followed up by face-to-face or by records only.

During the four-year follow-up, we identified a total of 1,614 stroke cases, including 1,156 ischemic, 416 intracerebral hemorrhage, and 42 subarachnoid hemorrhage. Table 1 summarizes baseline participants’ characteristics according to quintile categories of serum non-HDLC concentration. The median value of non-HDLC from the lowest quintile to the highest quintile at baseline was 87.0 mg, 112.5 mg, 130.3 mg, 149.3 mg, and 179.8 mg, respectively. We found that there were significant differences in age, BMI, SBP, DBP, concentrations of serum TC and HDLC, and triglyceride concentration among groups. These variables, with an exception of HDLC, tended to be increased in the higher non-HDLC quintile groups, whereas only the serum HDLC levels were lower in the higher quintile groups. Compared to the lower non-HDLC quintile groups, there were more males, daily smokers, daily drinkers and a larger proportion of participants with inactive physical activity, hypertension, and diabetes in the higher non-HDLC quintile groups.

**Table 1:** Characteristics of Participants According to Quintile Groups of Non-High-Density Lipoprotein Cholesterol at baseline.

	Quintile*					**P value**
	**Q1**	**Q2**	**Q3**	**Q4**	**Q5**	
Median non-HDLC, mg/dl	87.0	112.5	130.3	149.3	179.8	<0.01
Number of persons	19180	19230	19083	19235	19188	<0.01
Male,%	79.3	78.0	79.6	80.3	80.9	<0.01
Age, year	51.0	51.2	51.3	51.8	52.3	<0.01
Smoking,%						
never	63.7	64.1	62.1	57.3	55.3	<0.01
former	5.2	4.9	4.9	5.7	6.0	<0.01
occasional	3.8	3.3	3.6	3.7	3.5	<0.01
daily	27.3	27.7	29.4	33.4	35.4	<0.01
Drinking,%						
never	61.4	62.5	61.4	56.0	54.6	<0.01
former	3.6	3.1	3.3	3.7	3.7	<0.01
occasional	19.3	19.1	18.9	20.7	19.4	<0.01
daily	15.8	15.3	16.4	19.6	22.4	<0.01
Physical activity,%						
inactive	7.3	7.6	8.2	9.7	10.5	<0.01
moderate	79.7	78.5	78.1	74.2	71.2	<0.01
very	12.9	13.9	13.7	16.1	18.3	<0.01
Education,%						
illiteracy or primary school	10.5	9.0	9.1	10.2	11.8	<0.01
middle school	80.5	83.5	84.6	83.3	82.6	<0.01
≥college	9.0	7.5	6.2	6.5	5.6	<0.01
Hypertension,%	40.6	40.8	43.4	44.7	49.5	<0.01
Diabetes,%	7.3	7.3	8.0	9.8	13.5	<0.01
Body mass index, kg/m^2^	24.1	24.5	24.8	25.2	25.5	<0.01
Systolic blood pressure, mmHg	125.0	126.7	129.3	130.0	130.0	<0.01
Diastolic blood pressure, mmHg	80.0	80.0	80.0	80.7	82.0	<0.01
Fasting blood glucose, mg/dl	89.6	90.5	91.9	93.5	95.9	<0.01
Total cholesterol, mg/dl	146.2	170.5	189.1	206.9	239.8	<0.01
HDLC, mg/dl	61.5	58.4	58.0	56.5	56.8	<0.01
Triglyceride, mg/dl	95.7	78.0	79.6	80.3	80.9	<0.01

Abbreviation: HDLC = high-density lipoprotein cholesterol.

* Q1 represents the lowest fifth of the data (1-20%), Q2 represents the second lowest fifth (21-40%), Q3 represents the middle fifth (41-60%), Q4 represents the second highest fifth (61-80%), and Q5 represents the highest fifth (81-100%).

### Serum non-HDLC is an independent risk factor for total and ischemic stroke

Multivariate analysis in Table 2 showed that serum non-HDLC level was an independent risk factor for both total stroke and ischemic stroke (for total stroke, HR=1.08, 95% CI, 1.03-1.12; for ischemic stroke, HR=1.10, 95% CI, 1.05-1.16) (Table 2). The HR for non-HDLC refers to the increase per each 20 mg/dl. Other factors including age, male, daily smoking, BMI, hypertension, and diabetes mellitus were independent risk factors for ischemic stroke, and the associated HRs with 95% CIs were 1.06 (1.05-1.06), 1.76 (1.41-2.19), 1.59 (1.35-1.88), 1.03 (1.015-1.05), 2.10 (1.82-2.42), 1.79 (1.54-2.09), respectively. Occasional or daily drinking is a protective factor for ischemic stroke with an HR of 0.65 (0.53-0.81) and 0.76 (0.63-0.92), respectively (Table 2). Serum non-HDLC level was not an independent risk factor for intracerebral hemorrhage stroke (HR=0.83, 95% CI, 0.66-1.05) and subarachnoid hemorrhage (HR=1.03, 95% CI, 0.96-1.10) (Table 2).

**Table 2:** Risk Factors for Ischemic Stroke and Total Stroke.

	Adjusted HR*(95% CI)			
**Variable**	**Total stroke**	**ICH stroke**	**Ischemic stroke**	**SAH stroke**
Male	1.52 (1.28-1.81)^†^	1.22 (0.89-1.68)	1.76 (1.41-2.19)^†^	0.54 (0.22-1.29)
Age^‡^	1.05 (1.05-1.06)^†^	1.04 (1.03-1.05)^†^	1.06 (1.05-1.06)^†^	1.03 (0.99-1.06)
Smoking				
never	1(reference)	1(reference)	1(reference)	1(reference)
former	1.07 (0.85-1.34)	1.46 (0.96-2.24)	0.95 (0.72-1.25)	0.96 (0.25-3.71)
occasional	1.39 (1.02-1.90)^†^	1.47 (0.83-2.60)	1.40 (0.96-2.04)	0.60 (0.08-4.75)
daily	1.43 (1.24-1.65)^†^	1.09 (0.81-1.47)	1.59 (1.35-1.88)^†^	0.88 (0.36-2.14)
Drinking				
never	1(reference)	1(reference)	1(reference)	1(reference)
former	1.10 (0.87-1.40)	0.84 (0.49-1.45)	1.16 (0.88-1.52)	1.90 (0.38-9.51)
occasional	0.72 (0.60-0.86)^†^	0.81 (0.57-1.15)	0.65 (0.53-0.81)^†^	2.09 (0.82-5.31)
daily	0.81 (0.69-0.95)^†^	0.92 (0.66-1.28)	0.76 (0.63-0.92)^†^	1.99 (0.71-5.57)
Physical activity				
inactive	1(reference)	1(reference)	1(reference)	1(reference)
moderate	1.20 (0.97-1.48)	1.27 (0.82-1.97)	1.16(0.91-1.49	1.48 (0.44-4.99)
very	0.88 (0.70-1.12)	0.99 (0.61-1.64)	0.86 (0.65-1.13)	0.61 (0.15-2.88)
Hypertension	2.23 (1.97-2.51)^†^	2.66 (2.10-3.38)^†^	2.10 (1.82-2.42)^†^	1.87 (0.93-3.77)
Diabetes	1.57 (1.37-1.80)^†^	1.06 (0.78-1.44)	1.79 (1.54-2.09)^†^	0.95 (0.33-2.72)
Body mass index	1.03 (1.01-1.04)^†^	1.01 (0.98-1.04)	1.03 (1.01-1.05)^†^	1.08 (0.99-1.17)
Non-HDLC^§^	1.08 (1.03-1.12)^†^	1.03 (0.96-1.10)	1.10 (1.05-1.16)^†^	0.83 (0.66-1.05)

HRs, hazard ratios; CI, confidence interval; ICH, intracerebral hemorrhage; SAH, subarachnoid hemorrhage; Non-HDLC, non-high-density lipoprotein cholesterol

* Adjusted potential confounders included age, sex, body mass index, hypertension, diabetes, triglycerides, high-density lipoprotein cholesterol, smoking status, drinking status, physical activity status; stratified by hospitals.

^†^ P<0.05

^‡^ the HR for age refers to the increase per year.

^§^ the HR for non-HDLC refers to the increase per each 20 mg/dl.

### Higher serum non-HDLC is associated with increased risks of total and ischemic stroke independent of other potential confounding factors

As demonstrated in Table 3, serum non-HDLC levels were associated linearly with incidence of total stroke (P trend < 0.01) and ischemic stroke (P trend < 0.01). These associations were not changed after adjusting for potentially confounding factors (Table 3). Compared to the first quintile, the incidences of total stroke and ischemic stroke were significantly higher in the fourth (total stroke: HR=1.33, 95% CI, 1.12-1.59; ischemic stroke, HR=1.343, 95% CI, 1.09-1.66) and fifth quintiles (total stroke: HR=1.36, 95% CI, 1.15-1.62; ischemic stroke: HR=1.53, 95% CI, 1.24-1.88) after adjusting for the confounding factors. We did not find significant associations between serum non-HDLC cholesterol levels and incidences of intracerebral hemorrhage (P trend = 0.45) and subarachnoid hemorrhage (P trend = 0.12) (Table 3).

**Table 3:** Hazard Ratios (HRs) of Stroke According to Quintile Categories of Non-High-Density Lipoprotein Cholesterol.

	**Quintile (median non-high-density lipoprotein cholesterol, mg/dl)**						**P trend**
	**Q1(87.0)**	**Q2(112.5)**	**Q3(130.3)**	**Q4(149.3)**	**Q5(179.8)**		
Total stroke							
Incidence case (%)	261 (1.36)	298 (1.55)	297 (1.56)	361 (1.88)	397 (2.07)		
Crude HR (95% CI)	1	1.15 (0.97-1.35)	1.17 (0.99-1.38)	1.42 (1.21-1.67)	1.60 (1.37-1.88)	<0.01	
Adjusted HR^*^(95% CI)	1	1.19 (0.99-1.42)	1.16 (0.97-1.39)	1.33 (1.12-1.59)	1.36 (1.15-1.62)	<0.01	
Ischemic stroke							
Incidence case (%)	179 (0.93)	206 (1.07)	208 (1.09)	256 (1.33)	307 (1.69)		
Crude HR (95% CI)	1	1.15 (0.94-1.40)	1.19 (0.97-1.45)	1.50 (1.20-1.77)	1.81 (1.50-2.18)	<0.01	
Adjusted HR^*^(95% CI)	1	1.17 (0.95-1.45)	1.20 (0.97-1.48)	1.34 (1.09-1.66)	1.53 (1.24-1.88)	<0.01	
Intracerebral hemorrhage stroke							
Incidence case (%)	72 (0.38)	80 (0.42)	86 (0.45)	94 (0.49)	84 (0.44)		
Crude HR (95% CI)	1	1.14 (0.83-1.57)	1.24 (0.91-1.71)	1.38 (1.01-1.89)	1.26 (0.91-1.74)	0.08	
Adjusted HR^*^(95% CI)	1	1.21 (0.87-1.69)	1.19 (0.85-1.66)	1.36 (0.98-1.89)	1.18 (0.78-1.55)	0.45	
Subarachnoid hemorrhage stroke							
Incidence case (%)	16 (0.05)	12 (0.06)	3 (0.02)	11 (0.06)	6 (0.03)		
Crude HR (95% CI)	1	1.07 (0.56-2.49)	0.27 (0.07-0.97)	0.88 (0.37-2.11)	0.48 (0.17-1.34)	0.14	
Adjusted HR^*^(95% CI)	1	1.27 (0.51-3.15)	0.30 (0.08-1.15)	0.93 (0.36-2.39)	0.46 (0.15-1.39)	0.12	

* Adjusted potential confounders included age, sex, body mass index, hypertension, diabetes, triglycerides, high-density lipoprotein cholesterol, smoking status, drinking status, physical activity status; stratified by hospitals.

In order to determine the effect of potential confounding factors on the significant association between serum non-HDLC level and stroke incidences shown in Table 4, we further performed analysis of the data stratified by different risk factors. As shown in Table 4, gender, age, hypertension, diabetes, smoking and BMI did not affect the association between serum non-HDLC concentration and ischemic stroke (P value for each interaction was 0.73,0.96,0.47,0.77,0.12,0.84, respectively), strongly suggesting that non-HDLC level in serum is an independent risk factor for incidence of ischemic stroke.

**Table 4:** Adjusted Hazard ratios^*^ with 95% confidence intervals of Ischemic Stroke According to Quintile Categories of Non-High-Density Lipoprotein Cholesterol Levels, Stratified by Selected Risk Factors.

	**Quintile (median non-high-density lipoprotein cholesterol, mg/dl)**					**P trend**	**P for interaction**
	**Q1(87.0)**	**Q2(112.5)**	**Q3(130.3)**	**Q4(149.3)**	**Q5(179.8)**		
Gender							
Female	1	1.02 (0.50-2.09)	0.83 (0.39-1.75)	1.48 (0.77-2.86)	1.46 (0.76-2.81)	0.09	0.73
Male	1	1.16 (0.93-1.46)	1.22 (0.97-1.52)	1.32 (1.06-1.64)	1.54 (1.25-1.91)	<0.01	0.73
Age, years							
<60	1	1.25 (0.90-1.73)	1.26 (0.91-1.73)	1.43 (1.05-1.96)	1.65 (1.22-2.24)	<0.01	0.96
≥60	1	1.07 (0.80-1.42)	1.11 (0.83-1.48)	1.24 (0.94-1.65)	1.40 (1.06-1.85)	<0.01	0.96
Hypertension							
Yes	1	1.02 (0.79-1.31)	1.05 (0.82-1.35)	1.15 (0.90-1.47)	1.34 (1.06-1.70)	<0.01	0.47
No		1.56 (1.04-2.35)	1.58 (1.05-2.39)	1.94 (1.30-2.89)	2.14 (1.44-3.19)	<0.01	0.47
Diabetes							
Yes	1	0.86 (0.50-1.47)	1.12 (0.68-1.84)	1.28 (0.80-2.04)	1.56 (1.00-2.42)	<0.01	0.77
No	1	1.21 (0.96-1.53)	1.19 (0.94-1.51)	1.35 (1.07-1.70)	1.51 (1.20-1.90)	<0.01	0.77
BMI, kg/m^2^							
<25	1	1.35 (0.99-1.83)	1.28 (0.94-1.76)	1.45 (1.07-1.98)	1.60 (1.18-2.19)	<0.01	0.84
≥25	1	0.97 (0.72-1.31)	1.06 (0.79-1.42)	1.20 (0.91-1.59)	1.42 (1.08-1.86)	<0.01	0.84
Current smoking							
Yes	1	1.14 (0.79-1.65)	1.42 (0.99-2.01)	1.38 (0.98-1.96)	1.37 (0.97-1.94)	0.05	0.12
No	1	1.28 (0.97-1.69)	1.08 (0.81-1.44)	1.41 (1.07-1.86)	1.70 (1.30-2.23)	<0.01	0.12

* Adjusted potential confounders included age, sex, body mass index, hypertension, diabetes, triglycerides, high-density lipoprotein cholesterol, smoking status, drinking status, physical activity status; stratified by hospitals

## Discussion

The results of the present large prospective study, based on a community population in China with greater than 96,000 participants, suggested that serum non-HDLC level is an independent risk factor for stroke and one of its subtypes, ischemic stroke. Our data identified a positive association between serum non-HDLC level and the incidences of total stroke and ischemic stroke.

Although the positive association of serum non-HDLC level with the risk of coronary heart diseases has been well documented [5,16,17],, information about the relationship between serum non-HDLC and stroke remains limited and controversial. A study by Ren et al. reported that higher serum non-HDLC levels were associated with the increased risk for ischemic stroke in a mid-aged Chinese population [11]. In contrast, some prospective studies, independently conducted in Japanese adult population did not find a significant association between serum non-HDLC and stroke incidences. Specifically, the Suita study by Okamura et al. indicated that the incidence of cerebral infarction stroke was not associated with serum non-HDLC level in either men or women [9]; The Japan Arteriosclerosis Longitudinal Study – Existing Cohorts Combine (JALS-ECC) study reported that serum non-HDLC concentration was not significantly associated with the risk of any of the stroke subtypes [10]. In the present report, we conducted a community-based, large prospective study by enrolling close to 1000,000 participants, and showed that the serum non-HDLC level is an independent risk factor for total and ischemic stroke and significantly associated with their incidences, which was not affected by potential confounding factors such as gender, age, sex and BMI. Our results are consistent with the study by Ren et al. and substantially extend their observations. Possible reasons to explain the discrepancies between our study and the two studies on Japanese population may be that ischemic stroke is a heterogeneous syndrome in which many different pathways can lead to the same clinical presentations. The classical pathogenesis for ischemic stroke have been defined as large-artery atherosclerotic infarction that may be caused by extracranial or intracranial artery atherosclerotic infarction, embolism from a cardiac source, small-vessel disease, dissection, hypercoagulable states, sickle cell disease, and other unidentified causes [18]. Serum TC or LDLC concentrations were associated with atherothrombotic infarction, but not with lacunar infarction that was usually caused by non-atherosclerotic disease of small diameter penetrating arteries [19,20]. The positive association between serum non-HDLC concentration and ischemic stroke in Chinese populations could partially be explained by the high frequency of atherothrombotic infarction. As some studies have indicated, there are more lacunar strokes than atherothrombotic infarctions Japanese adult population compared to Chinese or American adults [21–25]. Furthermore, non-HDLC is composed of several atherogenic lipoproteins, including VLDL, LDL, IDL and lipoprotein (a), and each of them is related to atherosclerosis. Thus, it is reasonable that there is a positive association between serum non-HDLC concentration and the risk of ischemic stroke in Chinese adults.

We also analyzed the relationship between the serum non-HDLC level and occurrence of hemorrhage stroke subtype. Our study reported a higher proportion of hemorrhagic stroke than those reported in Western countries, which is consistent with some previous studies conducted in China where the proportion of hemorrhagic strokes was reported to be 17.1% to 55.4% [26,27], higher than the western populations (6.5% to 19.6%) [28]. The difference might be due to the uncontrolled hypertension and higher prevalence of smoking in China. We did not find significant relationship between the serum non-HDLC level and the risk of intracerebral hemorrhage or subarachnoid hemorrhage. Previous reports about the association between the risk of hemorrhagic stroke and cholesterol have focused on LDL cholesterol and HDLC, but not non-HDLC, and their conclusions were inconsistent. Most of these studies demonstrated an association between low serum cholesterol levels and the risk of hemorrhagic stroke, such as the ARIC-CHS study [29] and the study by Cui et al [30]. However, in the Korean Medical Insurance Corporation Study, low serum cholesterol concentration did not increase the risk of intracerebral hemorrhage [31]. With respect to non-HDLC, the data describing the relationship between serum non-HDLC level and the risk of hemorrhagic stroke are also controversial. The JALS-ECC study indicated that the risk of hemorrhagic stroke was not associated with serum non-HDLC level [10]. In contrast, Ren et al reported that compared to the lowest quintile group of non-HDLC, the risk of hemorrhagic stroke was not markedly declined in the top quintile group [11], which was consistent with our results. We were aware that compared to ischemic stroke the numbers of intracerebral and subarachnoid hemorrhage cases in this study were small, which is insufficient to determine if there is a positive, negative or no association between these stroke subtypes and non-HDL-C. The wide CIs are consistent with all three possibilities. Therefore, future study with larger hemorrhagic case number is needed to further address this question.

Compared to previous reports, our study demonstrated unique strengths and advantages. First, our data were generated from a prospective cohort study with a very large sample size (~ 1000,000 adults). Of importance, the Chinese adult participants enrolled in our study showed a similar baseline participants’ distribution as other epidemiological studies in China [32,33]. Additionally, individuals who took lipid-lowering medications were excluded from our study, making the serum lipid level in our cohort participants highly close to the normal population. With such large sample size and several years of accurate follow-up, we were able to identify that the serum non-HDLC level is an independent risk factor for stroke and associated with increased incidences of total and ischemic stroke. A few limitations of this study need to be acknowledged. Firstly, it is possible that the association between serum non-HDLC concentration and the risk for stroke is underestimated because of the single measurement of serum non-HDLC level at the baseline survey. Secondly, we should be careful to translate our observation to general Chinese population due to selection of participants in our study. The participants enrolled in our study were mainly the mining employees of the Kailuan group with a significantly higher ratio of male to female (80% to 20%) than the general Chinese population. Thirdly, we analyzed the effect of potential confounding factors on the association between serum non-HDLC levels and stroke incidences. Unfortunately one of the two groups for each risk factor examined has very wide CIs possibly due to small numbers. Therefore, we are aware that this component of the analyses is underpowered and might be only suggestive of, rather than directly demonstrating, no interactions or effect modification of these confounding factors. Lastly, the reported association between non-HDLC and risk of stroke may be further underestimated due to possible effect of silent infarction, which was not assessed in our study. Silent infarction has an estimated prevalence of 6%-28% and the prevalence is higher in elder generations [34,35].

## Conclusions

The present large prospective cohort study based on a Chinese community population showed that the serum non-HDLC is an impendent risk factor for stroke and its certain subtypes, and that high serum non-HDLC concentration is associated with the increased risk for total stroke and ischemic stroke, but not for intracerebral hemorrhage and subarachnoid hemorrhage. As a non-expensive and easily tested biomarker, non-HDLC may represent an important alternative screening marker for primary prevention of ischemic stroke among Chinese adults.
